# Supplementary material for: Depression, Anxiety, and Health-Related Quality of Life in Adults with Rheumatoid Arthritis: Findings from a National Survey
Source: J Clin Med. 2025 Nov 9;14(22):7940. doi: 10.3390/jcm14227940 (PMC12653145; doi:10.3390/jcm14227940)
Supplement: Supplementary file 1 [file jcm-14-07940-s001.zip › jcm-3957069-supplementary.pdf]

Supplementary Tables: Effect Sizes, Test Statistics, and Post-hoc Results

Table S1. Characteristics of the Study Sample, Number and Row Percentage of Characteristics by Rheumatoid Arthritis Groups.

	Total Sample		RA Only		RA & Dep		RA & Anx		RA & Dep & Anx					
	N	Wt.%	N	Wt.%	N	Wt.%	N	Wt.%	N	Wt.%	χ² (df)	Effect size	95 % CI	p-value
**All**	2324	100.0	1610	69.6	268	11.8	258	10.7	188	7.9				
**Age in years**														
22-39	106	5.7	59	58.7	13	12.7	19	15.5	15	13.1	$\chi^2(9) = 29.01$	0.065	0.024-0.105	<0.0001
40-49	190	8.6	112	57.8	23	11.6	24	13.3	31	17.3				
50-64	861	38.4	573	68.2	119	14.3	91	8.9	78	8.6				
>64	1167	47.3	866	74.2	113	9.8	124	11.0	64	5.0				
**Gender**														
Women	1670	68.1	1091	65.6	209	12.7	213	12.5	157	9.2	$\chi^2(3) = 17.15$	0.065	0.024–0.105	<0.0001
Men	654	31.9	519	78.2	59	10.0	45	6.8	31	5.1				
**Race/ethnicity**														
White	1241	63.8	838	67.2	138	11.4	142	11.8	123	9.6	$\chi^2(9) = 16.28$	0.048	0.007–0.088	0.061
African American	498	15.5	362	75.2	53	10.5	60	10.4	23	3.8				
Latino	434	13.3	294	71.6	58	13.6	46	8.3	36	6.5				
others	151	7.4	116	74.9	19	14.7	10	5.7	6	4.6				
**Marital status**														
Married	1041	51.6	773	73.3	112	11.4	91	8.9	65	6.4	$\chi^2(6) = 10.64$	0.043	0.002–0.084	0.100
Widow/Sep/Div	1016	39.3	667	66.8	125	11.5	132	12.3	92	9.4				
Never married	267	9.2	170	61.1	31	15.6	35	13.1	31	10.2				
**Education level**														
< High school	242	6.9	158	64.2	41	21.4	24	8.2	19	6.2	$\chi^2(6) = 12.07$	0.046	0.005-0.086	0.060
High school	323	11.1	201	62.1	46	15.6	54	13.2	22	9.1				
> High school	1719	80.7	1222	71.1	175	10.5	178	10.5	144	7.9				
**Region**														
Northeast	327	15.7	217	64.6	38	12.8	31	10.4	41	12.2	$\chi^2(9) = 20.83$	0.055	0.014–0.095	0.013
Mid-west	440	19.7	322	74.9	40	9.9	41	6.8	37	8.5				
South	1046	43.1	694	66.1	131	12.4	140	13.8	81	7.7				
West	511	21.5	377	75.4	59	11.9	46	8.1	29	4.7				

Table S1. Characteristics of the Study Sample, Number and Row Percentage of Characteristics by Rheumatoid Arthritis Groups.

	Total Sample		RA Only		RA & Dep		RA & Anx		RA & Dep & Anx					
	N	Wt.%	N	Wt.%	N	Wt.%	N	Wt.%	N	Wt.%	χ² (df)	Effect size	95 % CI	p-value
**Employment**														
Employed	666	33.7	500	75.5	55	8.8	65	8.8	46	6.9	$\chi^2(3) = 9.77$	0.049	0.008–0.089	0.021
Not employed	1658	66.3	1110	66.6	213	13.4	193	11.6	142	8.4				
**Poverty status**														
Poor	580	16.7	361	61.7	89	15.6	75	12.9	55	9.9	$\chi^2(9) = 19.46$	0.053	0.012–0.093	0.022
Near Poor	592	23.1	397	68.3	72	12.5	74	10.6	49	8.6				
Middle Income	560	25.6	387	66.9	56	12.0	71	13.3	46	7.8				
High Income	592	34.6	465	76.3	51	9.5	38	7.7	38	6.6				
**Health Insurance**														
Private	1013	52.1	760	74.3	88	9.3	98	9.6	67	6.7	$\chi^2(6) = 16.10$	0.053	0.012–0.093	0.013
Public	1271	46.3	822	64.4	177	14.8	155	11.7	117	9.1				
Uninsured	40	1.6	28	68.3	3	6.6	5	15.1	4	9.9				
**Rx Insurance**														
Rx insurance	729	38.9	545	74.1	64	9.6	67	8.9	53	7.4	$\chi^2(3) = 6.27$	0.039	-0.002–0.080	0.099
No Rx insurance	1595	61.1	1065	66.7	204	13.2	191	11.8	135	8.2				
**General health**														
Excellent/vgood	501	24.4	392	75.7	49	10.7	45	11.0	15	2.6	$\chi^2(6) = 45.70$	0.09	0.050–0.130	<0.0001
Good	799	34.7	592	74.2	76	10.0	89	10.9	42	4.9				
Fair/poor	1024	40.9	626	62.0	143	14.1	124	10.3	131	13.6				
**Physical activity**														
3/week	906	40.6	636	69.7	89	8.8	119	14.0	62	7.4	$\chi^2(3) = 16.18$	0.063	0.022–0.103	0.001
No exercise	1412	59.2	968	69.4	179	13.9	139	8.4	126	8.3				
**Heart**														
Yes	446	20.1	291	65.5	58	13.0	53	12.4	44	9.0	$\chi^2(3) = 1.91$	0.022	-0.082-0.091	0.590
No	1878	79.9	1319	70.6	210	11.5	205	10.2	144	7.6				
**Hypertension**														
Yes	1355	54.3	913	66.8	170	13.5	163	11.6	109	8.1	$\chi^2(3) = 5.80$	0.038	-0.003–0.079	0.122
No	969	45.7	697	72.9	98	9.8	95	9.6	79	7.7				

Supplementary Tables: Effect Sizes, Test Statistics, and Post-hoc Results

Table S1. Characteristics of the Study Sample, Number and Row Percentage of Characteristics by Rheumatoid Arthritis Groups.

	Total Sample		RA Only		RA & Dep		RA & Anx		RA & Dep & Anx					
	**N**	**Wt.%**	**N**	**Wt.%**	**N**	**Wt.%**	**N**	**Wt.%**	**N**	**Wt.%**	**χ^2 (df)**	**Effect size**	**95 % CI**	**p-value**
**Diabetes**														
Yes	583	21.6	402	71.9	75	11.8	52	8.1	54	8.1	$\chi^2(3) = 2.79$	0.026	-0.015–0.067	0.424
No	1741	78.4	1208	69.0	193	11.8	206	11.4	134	7.9				
**Hyperlipidemia**														
Yes	1028	43.7	217	64.2	47	15.1	34	9.8	37	10.9	$\chi^2(3) = 15.16$	0.061	0.020–0.101	0.002
No	1296	56.3	1393	70.6	221	11.2	224	10.8	151	7.4				
**Asthma**														
Yes	410	17.1	248	59.8	57	15.3	48	10.4	57	14.5	$\chi^2(3) = 15.56$	0.062	0.021–0.102	0.001
No	1914	82.9	1362	71.6	211	11.1	210	10.7	131	6.5				
**COPD**														
Yes	277	10.7	154	56.5	36	13.2	43	13.0	44	17.3	$\chi^2(3) = 24.37$	0.078	0.037–0.118	<0.0001
No	2047	89.3	1456	71.2	232	11.7	215	10.4	144	6.8				
**Osteoarthritis**														
Yes	335	15.3	679	65.5	128	13.2	142	14.2	79	7.1	$\chi^2(3) = 4.91$	0.035	-0.006–0.076	0.170
No	1989	84.7	931	72.8	140	10.8	116	7.9	109	8.5				
**GERD**														
Yes	499	20.6	298	60.2	69	14.9	63	11.6	69	13.3	$\chi^2(3) = 17.40$	0.066	0.025–0.106	<0.0001
No	1825	79.4	1312	72.0	199	11.0	195	10.4	119	6.5				
**Cancer**														
Yes	238	10.0	166	66.9	29	12.5	21	11.1	22	9.5	$\chi^2(3) = 0.74$	0.014	-0.027–0.055	0.863
No	2086	90.0	1444	69.9	239	11.8	237	10.6	166	7.7				

P value represents baseline differences between Rheumatoid Arthritis groups and chi-square tests. Anx: Anxiety; COPD: Chronic obstructive pulmonary disease; Dep: Depression; df: degrees of freedom; GERD: Gastro Esophageal reflux disease; Wt: weighted; RA: Rheumatoid arthritis; Rx: Medication. Effect size (Φ or V): Yule's Φ (2×2) and Cramer's V (>2×2) indicate effect size strength. Benchmarks (Cohen, 1988): small = 0.10 ∣ medium = 0.30 ∣ large = 0.50.

Supplementary Tables: Effect Sizes, Test Statistics, and Post-hoc Results

Table S2. Weighted Means and Standard Error Health-related Quality of Life Scores by Rheumatoid Arthritis Groups.

	**Total Sample**		**RAs Only**		**RA & Dep**		**RA & Anxiety**		**RA & Dep & Anxiety**			**Effect Size Measure**	**Test Statistics Reported**
	**Mean**	**SD**	**Mean**	**SE**	**Mean**	**SE**	**Mean**	**SE**	**Mean**	**SE**	**p-value**		
**HRQoL**													
**PCS**	36.16	12.23	38.53	0.46	35.50	1.05	36.23	1.21	33.99	1.26	<0.0001	Partial $\eta^2 = 0.0199$, $\omega^2 = 0.0189$	$F(3, 2320) = 15.79$, $p < 0.0001$
**MCS**	47.56	11.28	51.15	0.33	42.74	1.05	43.85	0.92	38.55	1.01	<0.0001	Partial $\eta^2 = 0.175$, $\omega^2 = 0.175$	$F(3, 2320) = 164.22$, $p < 0.0001$

Asterisks represent significant mean differences by Rheumatoid Arthritis groups using Anova MCS: Mental Component Summary; PCS: Physical Component Summary; SE: Standard Error; SD: Standard Deviation; Sig: Significance. Partial η^2: partial eta-squared; ω^2: omega-squared.

Table S3. Tukey's HSD Post Hoc Results.

Tukey's HSD Post Hoc Results — Physical Component Summary

Comparison	Mean Difference	95% CI	Tukey's t-statistic	df	p-value	Cohen's d	95% CI for d	Sig
RA only vs. RA + Depression	2.89	(0.834, 4.945)	t ≈ 3.08	2320	< 0.001	0.23	(0.110, 0.350)	***
RA only vs. RA + Anxiety	3.521	(1.431, 5.610)	t ≈ 3.58	2320	< 0.001	0.28	(0.150, 0.400)	***
RA only vs. RA + Dep & Anxiety	4.846	(2.445, 7.247)	t ≈ 4.56	2320	< 0.001	0.37	(0.240, 0.500)	***
RA + Depression – RA + Anxiety	0.631	(−2.086, 3.348)	t ≈ 0.65	2320	0.514	0.05	(−0.090, 0.190)	
RA + Depression vs. RA + Dep & Anxiety	1.956	(−1.008, 4.920)	t ≈ 2.00	2320	0.136	0.16	(−0.040, 0.280)	
RA + Anxiety vs. RA + Dep & Anxiety	1.325	(−1.662, 4.313)	t ≈ 1.35	2320	0.228	0.11	(−0.070, 0.250)	

Tukey's HSD Post Hoc Results — Mental Component Summary

Comparison	Mean Difference	95% CI	Tukey's t-statistic	df	p-value	Cohen's d	95% CI for d	Sig
RA only vs. RA + Depression	9.246	(7.507, 10.985)	t ≈ 9.03	2320	< 0.001	0.9	(0.770, 1.030)	***
RA only vs. RA + Anxiety	8.043	(6.275, 9.811)	t ≈ 8.35	2320	< 0.001	0.78	(0.660, 0.900)	***
RA only vs. RA + Dep & Anxiety	13.393	(11.361, 15.425)	t ≈ 12.60	2320	< 0.001	1.24	(1.100, 1.380)	***
RA + Anxiety – RA + Depression	1.203	(−1.096, 3.503)	t ≈ 1.15	2320	0.252	0.11	(−0.040, 0.260)	
RA + Dep & Anxiety vs. RA + Dep	4.147	(1.639, 6.656)	t ≈ 3.94	2320	< 0.001	0.37	(0.240, 0.500)	***
RA + Dep & Anxiety vs. RA + Anxiety	5.351	(2.823, 7.879)	t ≈ 5.22	2320	< 0.001	0.49	(0.360, 0.620)	***

Note. Tukey's HSD post hoc comparisons; Cohen's d thresholds: small = 0.2, medium = 0.5, large = 0.8 (Cohen, 1988).

Table S4. Intercept and Parameter Estimates from Adjusted Multivariate Linear Regressions on HRQoL among Adults with Rheumatoid Arthritis.

	Health-Related Quality of Life									
	Physical Component Summary					Mental Component Summary				
	β (Std.)	B (Unstd.)	SE	t	P value	β (Std.)	B (Unstd.)	SE	t	P value
**Hyperlipidemia Group**										
RA & depression	−0.17	−0.450	0.055	−8.18	< 0.001 ***	−0.32	−6.608	0.106	−62.29	< 0.001 ***
RA & anxiety	−0.19	−1.101	0.131	−8.38	< 0.001 ***	−0.33	−6.836	0.015	−443.30	< 0.001 ***
RA & depression & anxiety	0.01	0.071	0.143	0.5	0.62	−0.45	−9.766	0.116	−84.19	< 0.001 ***
RA only (Ref.)										
**Age in years**										
22-39	0.38	2.747	0.066	41.47	< 0.001 ***	−0.12	−2.462	0.046	−53.31	< 0.001 ***
40-49	0.1	0.598	0.051	11.85	< 0.001 ***	−0.10	−2.148	0.025	−86.92	< 0.001 ***
50-64	0.08	0.419	0.06	6.96	< 0.001 ***	−0.14	−2.767	0.029	−95.98	< 0.001 ***
>65 (Ref.)										
**Gender**										
Men	0.04	0.203	0.087	2.34	0.020 *	0.03	0.088	0.024	3.59	0.0004 **
women (Ref.)										
**Race/ethnicity**										
African American	0.08	0.494	0.037	13.25	< 0.001 ***	0.06	0.55	0.038	14.55	< 0.001 ***
Latino	0.2	2.055	0.052	39.34	< 0.001 ***	−0.09	−0.666	0.034	−19.64	< 0.001 ***
Others	0.18	1.93	0.007	267.78	< 0.001 ***	−0.22	−2.039	0.015	−134.72	< 0.001 ***
White (Ref.)										
**Marital status**										
Married	0.07	0.511	0.027	19.24	< 0.001 ***	0.08	1.015	0.016	64.67	< 0.001 ***
Widow/Sep/Divorce	−0.05	−0.209	0.042	−5.00	< 0.001 ***	0.09	1.177	0.011	107.01	< 0.001 ***
Never married (Ref.)										
**Education level**										
> High school	−0.08	−0.421	0.074	−5.71	< 0.001 ***	0.1	1.16	0.084	13.74	< 0.001 ***
High school	−0.01	−0.066	0.063	−1.05	0.297	−0.03	−0.153	0.061	−2.49	0.013 *
< High school (Ref.)										

Table S4. Intercept and Parameter Estimates from Adjusted Multivariate Linear Regressions on HRQoL among Adults with Rheumatoid Arthritis.

	Health-Related Quality of Life									
	Physical Component Summary					Mental Component Summary				
	β (Std.)	B (Unstd.)	SE	t	P value	β (Std.)	B (Unstd.)	SE	t	P value
**Region**										
Northeast	0.04	0.256	0.008	33.7	< 0.001 ***	−0.05	−0.475	0.012	−40.58	< 0.001 ***
Mid-west	0.15	2.138	0.019	114.3	< 0.001 ***	0.03	0.375	0.024	15.58	< 0.001 ***
South	−0.10	−0.965	0.059	−16.26	< 0.001 ***	−0.02	−0.173	0.008	−21.92	< 0.001 ***
West (Ref.)										
**Employment**										
Employed	0.25	4.439	0.042	105.07	< 0.001 ***	0.07	0.941	0.046	20.55	< 0.001 ***
Not employed (Ref.)										
**Poverty status**										
Poor	−0.30	−2.281	0.036	−63.81	< 0.001 ***	−0.20	−2.612	0.051	−51.21	< 0.001 ***
Near Poor	−0.33	−3.570	0.046	−78.01	< 0.001 ***	−0.17	−2.169	0.028	−76.13	< 0.001 ***
Middle Income	−0.28	−2.218	0.033	−66.23	< 0.001 ***	−0.10	−1.179	0.047	−25.12	< 0.001 ***
High Income (Ref.)										
**Rx Insurance**										
Rx insurance	−0.25	−1.322	0.009	−148.84	< 0.001 ***	−0.12	−2.139	0.038	−56.27	< 0.001 ***
No Rx insurance (Ref.)										
**General health**										
Excellent/very good	0.6	12.315	0.022	559.73	< 0.001 ***	0.26	5.118	0.026	196.84	< 0.001 ***
Good	0.35	6.91	0.067	102.95	< 0.001 ***	0.22	4.784	0.059	81.43	< 0.001 ***
Fair/poor (Ref.)										
**Physical activity**										
3/week	0.22	3.436	0.016	217.16	< 0.001 ***	0.09	1.252	0.041	30.24	< 0.001 ***
No exercise (Ref.)										
**Heart**										
Yes	−0.09	−0.968	0.048	−20.07	< 0.001 ***	−0.03	−0.198	0.077	−2.57	0.011 *

Table S4. Intercept and Parameter Estimates from Adjusted Multivariate Linear Regressions on HRQoL among Adults with Rheumatoid Arthritis.

	Health-Related Quality of Life									
	Physical Component Summary					Mental Component Summary				
	β (Std.)	B (Unstd.)	SE	t	P value	β (Std.)	B (Unstd.)	SE	t	P value
**Hypertension**										
Yes	−0.10	−1.274	0.06	−21.24	< 0.001 ***	0.02	0.187	0.024	7.88	< 0.001 ***
**Diabetes**										
Yes	−0.12	−1.584	0.022	−71.47	< 0.001 ***	−0.06	−0.599	0.009	−69.67	< 0.001 ***
**Asthma**										
Yes	−0.11	−1.199	0.015	−81.31	< 0.001 ***	−0.10	−1.487	0.036	−41.60	< 0.001 ***
**COPD**										
Yes	−0.09	−1.430	0.072	−19.85	< 0.001 ***	0.09	1.35	0.068	19.85	< 0.001 ***
**GERD**										
Yes	−0.08	−0.288	0.007	−38.36	< 0.001 ***	−0.04	−0.398	0.069	−5.75	< 0.001 ***
**Cancer**										
Yes	−0.01	−0.100	0.109	−0.92	0.359 ns	−0.02	−0.130	0.01	−12.70	< 0.001 ***

Asterisks denote statistical significance in parameter estimates from multivariate linear regressions on health-related quality of life. ***P <0.001; *0.01 < P <.05.

COPD: Chronic obstructive pulmonary disease; GERD: Gastro Esophageal reflux disease; RA: Rheumatoid arthritis; Rx: Medication; Ref: reference group; SE: Standard Error; Sig: Significance. Wid./Div./Sep.: widowed, divorced, and separated.

β (Std.): Standardized regression coefficient Beta (β); B (Unstd.): Unstandardized regression coefficient (B) .

Model fits PCS: $R^2 = 0.4121$; Cohen's $f^2 = 0.701$ (Large effect size).

Model fits MCS: $R^2 = 0.3125$; Cohen's $f^2 = 0.455$ (Large effect size).
